# The Clinic Handbook: A Quality Improvement Project

**DOI:** 10.7759/cureus.99355

**Published:** 2025-12-16

**Authors:** Success T Oyibo, Amy Carter

**Affiliations:** 1 Internal Medicine, Northern Care Alliance, Manchester, GBR; 2 Internal Medicine, Manchester Foundation Trust, Manchester, GBR

**Keywords:** clinic organisation, handbook implementation, improving trainee experience, internal medicine training, quality improvement in healthcare, quality improvement project (qip), quality improvement projects, quality improvement study

## Abstract

Background: Internal Medicine Trainees (IMTs) are required to gain outpatient experience by scheduling clinics in each rotation. Due to a lack of knowledge of who to contact and varied scheduling procedures across different hospitals, trainees often encountered difficulties arranging these clinics.

Aim: To improve the organisation and educational value of clinic weeks for IMTs through the development and implementation of a comprehensive, user-friendly Clinic Handbook.

Methods: We used a pre- and post-intervention study design and carried out quantitative and qualitative analyses of the responses. First, we distributed a baseline survey of IMTs to identify barriers to organising clinic weeks. Subsequently, we designed a Clinic Handbook by liaising with departmental secretaries and clinic administrative staff. The handbook was electronically distributed to IMTs. After 12 weeks, a follow-up survey was performed. Quantitative data were analysed using descriptive statistics by calculating and comparing mean Likert scores. Qualitative comments were reviewed thematically. This work was structured around a Plan-Do-Study-Act (PDSA) quality improvement framework.

Results: Fifteen trainees completed the baseline survey, and sixteen completed the post-intervention survey. Mean Likert scores (1=strongly disagree to 5=strongly agree) improved across key domains: ease of scheduling clinics (2.33 to 4.33), knowing who to contact in current specialty (3.06 to 4.58), knowing who to contact in other specialties (1.93 to 4.58), ability to plan clinics on time (2.60 to 4.33), and having no issues with other professionals/students already attending the clinics (2.73 to 3.83). All post-handbook survey respondents found the handbook useful and reported increased confidence in arranging clinics beyond their current speciality.

Conclusion: A low-cost, trainee-designed Clinic Handbook addressed system and information barriers to outpatient training and broadened access to speciality clinics. Embedding the handbook into induction and maintaining regular updates will sustain and scale these gains for trainees across the organisation and nationally.

## Introduction

The Internal Medicine Training (IMT) program requires most trainees to rotate between hospitals at least annually and between departments every four to six months; consequently, they are often expected to schedule clinics within unfamiliar institutions [1].

Outpatient clinic experience is a cornerstone of IMT in the United Kingdom, promoting continuity of care, effective management of chronic diseases, and multidisciplinary teamwork. The Royal College of Physicians’ IMT curriculum emphasises outpatient exposure to develop diagnostic reasoning and longitudinal patient management skills [2].

At this University Teaching Hospital, trainees are allocated a dedicated 'clinic week' every six months to support these objectives. However, many experienced challenges in planning their clinic weeks, including unclear contact personnel, limited access to clinic schedules, and competition for clinic space with other trainees. These obstacles created inequities between trainees and hindered the intended learning outcomes.

To address this, a quality improvement project (QIP) was conducted to design and implement a comprehensive 'Clinic Handbook' that consolidated key clinic information and simplified the clinic scheduling processes.

## Materials and methods

Study setting

This QIP was executed at a university teaching hospital, involving IMT Year 1 to IMT Year 2 trainees during the 2023-2024 training year. 

Study design and measures

We used a pre- and post-intervention survey design and performed both quantitative and qualitative analyses of the responses [3-5].

Data collection and analysis 

Quantitative survey responses across five key domains were summarised using means for Likert items. The Likert scale is free to use because it is a measurement technique rather than a proprietary instrument and was originally introduced by R. Likert in 1932 [6]. No copyright restrictions apply to creating or adapting Likert-type items. Qualitative feedback was thematically reviewed to identify recurring perceptions of usefulness and barriers. No inferential statistics were applied, in line with the project’s focus on quality improvement.

Patient and public involvement

Neither patients nor the public were involved in the design, conduct or reporting of our research. The IMT zonal administrator assisted with distributing our survey via email to all trainees working at our institution.

Theory of change

A theory of change was developed to describe how the introduction of the Clinic Handbook was expected to improve trainees’ ability to organise clinic weeks effectively [7]. We hypothesised that the main barriers to successful clinic week organisation were a lack of accessible information, unclear communication channels and variation in local processes between hospitals.

PDSA cycle

The intervention, the Clinic Handbook, was developed and implemented using the Plan-Do-Study-Act (PDSA) framework [8-11]. 

Plan

We developed an electronic baseline survey (Google Forms, Google LLC, Mountain View, CA) to identify barriers experienced by IMTs when organising their clinic week. Using a Likert scale, survey domains included: (i) ease of scheduling clinics, (ii) knowledge of who to contact within the trainee's current speciality, (iii) knowledge of who to contact outside the trainee’s current speciality, (iv) ability to arrange clinics in advance, and (v) Issues with over-attendance. We also collected free-text feedback to capture qualitative themes and specific suggestions.

Do

Survey findings informed the development of the ‘Clinic Handbook’. The handbook compiled clinic locations, times and named contacts for clinic booking (consultants and secretaries). The content was reviewed with departmental secretaries and outpatient departments to ensure accuracy and acceptability. The handbook was distributed electronically to IMTs and shared with educational supervisors.

Study

After 12 weeks of implementation, a follow-up survey using the same core Likert scale and domains was disseminated to IMTs. We compared pre- and post-intervention responses. Likert responses (Strongly Disagree - Strongly Agree) were numerically coded 1-5. Descriptive statistics were generated in Microsoft Excel (Microsoft Corp., Redmond, WA). We calculated mean scores for each domain before and after the intervention. We also undertook a thematic review of free-text responses to assess the perceived usefulness of the handbook and residual barriers.

Act

Based on the findings, we made recommendations to improve the clinic experience for IMTs, which included routine distribution of the handbook at trainee induction and to all trainee supervisors, and commitment to periodic handbook updates.

## Results

Quantitative findings

A total of 15 trainees participated in the pre-handbook survey, and 16 trainees participated in the post-handbook survey. Most of the post-handbook survey respondents (75%) had used the handbook to organise their clinics. We excluded four respondents who had not used the handbook from our analysis and used a 5-point Likert scale with scores ranging from 1 (Strongly Disagree) to 2 (Disagree), 3 (Neutral), 4 (Agree), and 5 (Strongly Agree) to assess the key domains as shown in Table 1. 

**Table 1:** Comparison of mean scores for key domains assessed in the pre- and post-handbook surveys Q1-Q5 = survey domains [6]

	Question/domain	Pre-handbook Mean	Post-handbook Mean
Q1.	It was easy to schedule clinics to attend during my clinic week.	2.33	4.33
Q2.	I knew who to get in touch with to schedule clinics in my current rotation speciality.	3.06	4.58
Q3.	I knew who to get in touch with to schedule clinics outside of my current rotation speciality.	1.93	4.58
Q4.	I was able to schedule clinics ahead of time.	2.60	4.33
Q5.	I had no issues with other professionals/students already attending the clinics.	2.73	3.83

**Figure 1:**
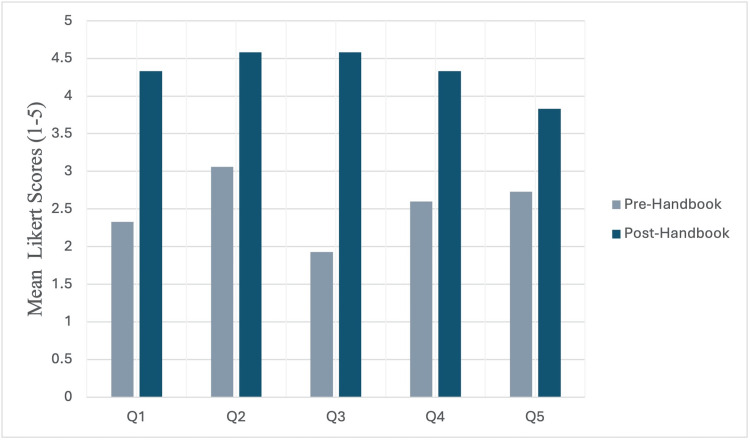
Mean Likert survey scores before and after distribution of the Clinic Handbook Q1-Q5 = survey domains [6]

Following the handbook distribution, Mean Likert survey scores increased across all five domains, indicating improved clarity and ease of organisation of clinics. The most significant improvements were observed in knowledge of who to contact outside the trainee’s current speciality and in overall ease of scheduling clinics, as shown in Figure 1. No negative consequences or unintended workload increases were reported.

Qualitative findings

We applied thematic analysis to qualitative feedback as described and used in similar studies [12,13]. Pre-handbook comments described significant difficulties scheduling clinics and identifying which sessions were suitable for IMTs. Common issues included uncertainty about whom to contact, how to arrange clinics and the location of various clinics. Some reported repeated unsuccessful enquiries to outpatient departments or secretaries. The competition for clinic spots with medical students and other trainees led to limited opportunities to participate or see their own patients.

Post-intervention, trainees provided positive feedback regarding the clinic handbook, describing it as a valuable and practical resource that simplified the process of organising clinics. They appreciated the effort involved and described the handbook as 'a lifesaver,' 'very helpful,' and 'a great way to know clinics outside my speciality.' Some reported that the handbook improved communication with appropriate secretaries and helped reduce scheduling conflicts.

Balancing measures and intervention fidelity

Balancing measures were considered to ensure that the introduction of the Clinic Handbook did not lead to unintended challenges for trainees, such as increased workload or scheduling difficulties [14,15]. The post-intervention survey included an open-ended comment inviting trainees to share their experiences of using the handbook and to comment on any positive or negative effects it had on their ability to arrange clinic weeks.

Intervention fidelity was evaluated by assessing whether trainees accessed and used the handbook as intended [16]. The majority of the post-handbook survey respondents had used the handbook to organise their clinic weeks, and all users found it helpful. These findings suggest that the intervention was implemented with high fidelity and was well integrated into routine trainee practice.

## Discussion

The findings from this quality improvement project provide strong support for the underlying theory of change [17,18]. The initial assumption, that a lack of accessible and structured information was a key barrier to clinic week organisation, was validated by the baseline survey, which revealed widespread uncertainty regarding clinic timetables, contacts, and booking procedures.

The introduction of the Clinic Handbook directly addressed these barriers by offering a centralised, easy-to-use resource, improving access to scheduling information and reducing administrative burden. Post-handbook feedback from trainees indicated that access to information was the primary driver of improvement, resulting in greater confidence, better planning, and more equitable participation in outpatient learning.

This project demonstrated that a simple, trainee-led intervention can meaningfully improve access to outpatient learning opportunities for IMTs. The increase in mean Likert scores across all domains supports the effectiveness of the handbook in organising clinics [19]. Qualitative feedback further reinforced that trainees experienced greater clarity on whom to contact, improved ability to plan, and fewer disruptions due to overbooked clinics.

Importantly, the intervention also addressed an issue of equity [20]. Before introducing the handbook, trainees who had previously worked in the institution or who received strong supervisory support were more confident in arranging clinic exposure, while newer trainees were disadvantaged. The handbook promoted a more consistent and inclusive approach to outpatient learning across all training levels. These findings align with broader postgraduate training priorities that emphasise protected clinic time and equitable access to diverse speciality experiences. Embedding structured guidance into trainee induction recognises that navigating administrative systems can itself be a barrier to learning and supports early engagement with outpatient learning.

The implementation of the Clinic Handbook was influenced by a combination of barriers and enabling factors [19]. Understanding these factors is essential for interpreting the outcomes of this project and for informing future scale-up efforts across other hospitals. 

The key barriers and facilitators identified through trainee feedback and project reflection are summarised in Table 2.

**Table 2:** Barriers and facilitators of implementing the handbook

Category	Description/relevance to the project
Barriers	
Variable trainee rotations	Frequent movement of IMTs between hospitals limited continuity and awareness of the handbook across successive cohorts.
Dependence on regular updates	The handbook’s usefulness depended on maintaining accurate clinic timetables and contact details, requiring ongoing review and administrative input.
Limited initial awareness	Some trainees were unaware of the handbook’s existence during the initial rollout, particularly those who already had their clinic week.
Facilitators	
Trainee-led design	The project was developed by IMTs who understood local barriers, increasing engagement, ownership, and practical relevance.
Low-cost, easy-to-distribute format	The digital format enabled rapid, cost-free dissemination across departments and specialities.
Alignment with curriculum goals	The handbook directly supported IMT curriculum objectives for outpatient experience, enhancing its educational legitimacy.
Positive trainee feedback	Qualitative feedback described the handbook as helpful and time-saving, reinforcing continued use.
Perceived relevance and usability	The handbook’s clear layout and concise content made it an accessible, user-friendly resource for trainees at all levels.

The success of the handbook was largely driven by its trainee-led design [21], low-cost digital format, and alignment with existing curriculum objectives. On the other hand, barriers should be addressed to optimise long-term adoption. Regarding sustainability, the handbook must be kept up to date: clinic schedules, locations, and consultant allocations inevitably evolve as consultants change jobs, necessitating periodic review with outpatient administration to maintain accuracy [22].

## Conclusions

The Clinic Handbook provided IMTs with an accessible, structured tool to organise clinics effectively. The project improved communication and enhanced the overall quality of training. Its trainee-led design, digital format, and minimal cost make the model adaptable for wider implementation across other hospital trusts and potentially other IMT programmes nationally. To ensure sustained benefits of this quality improvement project, we recommend that the postgraduate team formally incorporate the clinic handbook into the induction pack for all IMTS and share the clinic handbook with educational supervisors to ensure trainees receive consistent support in organising their clinic weeks. Trainee Representatives should regularly oversee the updating of the clinic handbook to reflect evolving clinic timetables, contact information, and service structures. Finally, consideration should be given to scaling and adapting this model across other hospitals to promote standardisation and best practice across the wider training program.
